# The complete chloroplast genome of *Rhododendron huadingense* (Ericaceae)

**DOI:** 10.1080/23802359.2022.2135403

**Published:** 2022-10-30

**Authors:** Ran An, Mingyue Niu, Xiongzhen Lou, Huahong Huang, Erpei Lin

**Affiliations:** a State Key Laboratory of Subtropical Silviculture, Zhejiang A&F University, Hangzhou, Zhejiang, China; b Huading Forestry Farm, Tiantai, Zhejiang, China

**Keywords:** *Rhododendron huadingense*, chloroplast genome, phylogenetic analysis, Ericaceae

## Abstract

*Rhododendron huadingense* is an important horticultural plant that belongs to the Ericaceae family. In this study, the chloroplast genome sequence of *R. huadingense* is reported. The chloroplast genome of *R. huadingense* was 198,952 bp in length and had an angiosperm-typical quadripartite structure with a large single-copy (LSC) region of 108,557 bp, a small single-copy (SSC) region of 53 bp, and two inverted repeat regions (IRs) of 45,171 bp. One hundred and thirteen unique genes including 79 protein-coding genes, 30 transfer RNA (tRNA) genes, and four ribosomal RNA (rRNA) genes were identified in the chloroplast genome. Further phylogenetic analysis revealed a close relationship between *R. huadingense* and *R. molle*. The complete chloroplast genome of *R. huadingense* provides valuable genetic information for the phylogeny, varieties breeding and sustainable utilization of this species.

*Rhododendron huadingense* B.Y Ding & Y.Y Fang 1990 was first discovered and named by Ding Bingyang etc in Huading Mountain, Tiantai County, Zhejiang Province, China in 1990 (Ding and Fang 1990). It is an endangered species and has been designated as a priority protected wild plant in Zhejiang Province due to its limited habitats and tiny population (Li et al. 2019). *R. huadingense* is unique in terms of hairiness, inflorescence type, fruit form, and seed size, all of which are important in phylogenetic investigations. Besides, it also has a high ornamental value due to its beautiful purple flowers (Ding et al. 1995). On the other hand, with the development of high throughput sequencing technology, it became much easier to sequence and obtain the chloroplast genomic sequences (Guo et al. 2018). To date, the chloroplast genomes of many species of *Rhododendron* have been reported, including *R. delavayi* (Liu et al. 2020), *R. platypodum* (Ma et al. 2021), and *R. molle* (Liu et al. 2021). In this study, the chloroplast genome sequencing of *R. huadingense* will be a great help to molecular identification as well as conservation strategy developing for this species.

Fresh and clean leaves of *R. huadingense* were collected from Taizhou, Zhejiang, China (29°15′27.49″N, 121°5′38.47″E, 850 m). A voucher specimen was deposited at the Herbarium of Hangzhou Botanical Garden with no. HZ041835 (contact person: Yahong Gao; email: 164314353@qq.com). According to the International Union for Conservation of Nature (IUCN) policy on endangered species research, the sample collection and the study were conducted with permission from Huading Forestry Farm, Tiantai, China. The total genomic DNA was extracted by using the modified CTAB protocol. DNA libraries were prepared with an insert size of 350 bp using NEBNext Ultra DNA Library Prep Kit. Pair-end sequencing (150 bp reads) was performed on the Illumina Hiseq 2500 Platform (Tianjin, China). In total, 46.1 GB clean data were generated and utilized for the chloroplast genome assembly using NOVOPlasty 4.3.1 (Dierckxsens et al. 2020). The entire genome sequence was annotated by CPGAVAS2 (Shi et al. 2019) and GeSeq (Tillich et al. 2017) with manual adjustments, using chloroplast genome of *R. griersonianum* (MT533181) as a reference. The genome and its annotated genes were submitted to NCBI under the accession number OM177184.

The chloroplast genome of *R. huadingense* was 198,952 bp in length and presented an intact circular structure with two inverted repeat regions (IRs), a small single-copy (SSC), and a large single-copy (LSC). The SSC and LSC regions were divided by two IR regions. The SSC was only composed of 53 base pairs, the LSC was 108,557 bp in length, and the IR region was 45,171 bp in length. The overall GC content of the chloroplast genome was 35.89%. The GC content of the two IR regions (36.63%) was higher than that of the LSC (35.29%) and SSC (5.66%). The relative high GC content was caused by rRNA and tRNA genes, which was also observed in other plants (He et al. 2016). The chloroplast genome encoded 113 unique genes, including 79 protein-coding genes, 30 tRNAs, and four rRNAs.

Microsatellite of the chloroplast genome was analyzed using the MIcroSAtellite (MISA) program (Beier et al. 2017). The parameters were set as follows: 1–10, 2–6, 3–5, 4–4, 5–3, and 6–3; the minimum distance between two SSRs was set to 100 bp. In this study, we detected 75 SSRs, which included 67 mononucleotides (A/T), four dinucleotides (AT/AT), two pentanucleotides (AAAAT/ATTTT, ACATG/ATGTC), and two hexanucleotide (AAGGGT/ACCCTT, AATATT/AATATT). Mononucleotides are the most frequently repeated type, accounting for 89.3% of the total SSR markers.

According to the previous studies (Liu et al. 2021; Zhou et al. 2021), the chloroplast genomes of 15 related species were chosen for the phylogenetic analysis. Among these species, four *Rhododendron* species and three *Vaccinium* species of Ericaceae were used to interpret the phylogenetic relationships between *R. huadingense* and other species of Ericaceae, and the other eight species were used as outgroups. The phylogenetic analysis was carried out based on the coding sequences (CDS) of 59 shared genes from chloroplast genomes of *R. huadingense* and 15 related species. The chloroplast genome sequences of these 15 species were obtained from GenBank. All sequences were aligned using MAFFT v7.490 (Nakamura et al. 2018). Then, based on alignment results, the best tree model was determined by using jModeltest (Posada 2008). The maximum-likelihood (ML) analyses were conducted using RAxML 8.0 (Stamatakis 2006). For ML analyses, the best-fit model (general time reversible) with gamma distribution (GTR + G) was used with 1000 bootstrap replicates. According to the phylogenetic analysis, the *Rhododendron* plants formed a monophyletic clade with 100% bootstrap value, and *R. huadingense* had the closest relationship with *R. molle* (Figure 1).

**Figure 1.**
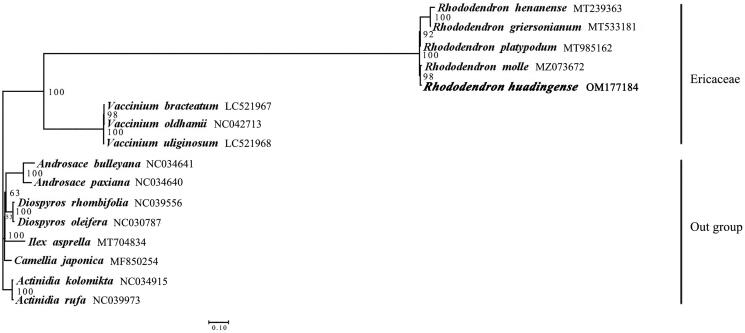
Maximum-likelihood (ML) phylogenetic tree based on the CDS of 59 shared genes from chloroplast genomes of *R. huadingense* and 15 other species. The support values are indicated at the branches. The accession number of GenBank for each species is listed in the figure.

## Data Availability

The data that support the findings of this study are openly available in GenBank of NCBI at https://www.ncbi.nlm.nih.gov under the accession number OM177184. The associated BioProject, SRA, and Bio-Sample numbers are PRJNA838786, SRR19256810, and SAMN28463168, respectively.
